# Dynamic Change of Intestinal Duplication in an Adult Patient: A Case Report and Literature Review

**DOI:** 10.1155/2012/297585

**Published:** 2012-06-20

**Authors:** Abhishek Shah, Jimin Du, Yutian Sun, Dianbo Cao

**Affiliations:** ^1^Department of Radiology, The First Hospital of Jilin University, Changchun 130021, China; ^2^Department of Pharmacy, China-Japan Union Hospital, Jilin University, Changchun 130021, China

## Abstract

Intestinal duplication in an adult is an uncommon congenital abnormality because only minority of cases present in adulthood. More than 80% of cases occur before the age of two years as an acute abdomen, bowel obstruction or other complications associated with it. Duplication has two types, either cystic or tubular. Here, we report a case of an adult who was diagnosed preoperatively on CT scan as tubular intestinal duplication. CT images showed change in the morphology of the cystic mass after one week of antibiotics administration. On histopathological analysis, the resected duplicated segment had esophageal epithelium in addition to the intestinal gland. So far, we found no report describing CT findings of dynamic change of ileal duplication in the English literatures.

## 1. Introduction

Gastrointestinal duplication is an uncommon congenital abnormality. More than 80% of cases present before the age of 2 years as an acute abdomen or bowel obstruction, and a minority may remain asymptomatic until adulthood [1]. It can occur anywhere throughout the alimentary tract from mouth to anus. Duplication of alimentary tract occurs with approximately 33% arising in the foregut, 56% in the midgut, and 11% in the hind gut. Ileal duplication, including cystic and tubular type, is the most common duplication with preponderance in males. Presentation may include vague abdominal pain, distention, and complications such as obstruction, bleeding, perforation, or malignancy. Diagnosis of duplication cysts, especially in the small intestine, may be difficult, attributed to inability to visualize with standard endoscopic investigation, so radiographic studies such as barium meal study, ultrasonography, and CT scan play an important role in establishing the correct diagnosis and guiding appropriate management. Timely discovery for ileal duplication can significantly lessen the prevalence of life-threatening complications in the future [2, 3]. Until now, there is no description about dynamic change of ileal duplication in such a short interval on CT examination. So, we report this patient with ileal duplication so as to improve our recognition for this infrequent disease entity.

## 2. Clinical Summary

**Figure 1:**
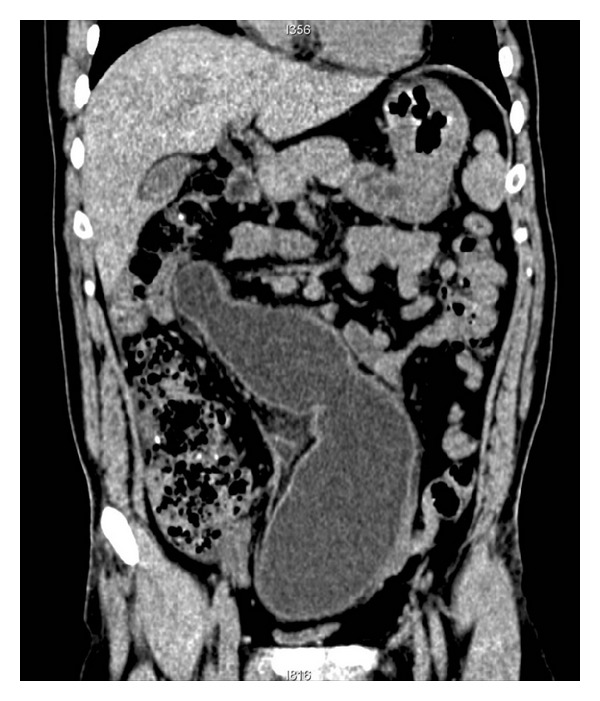
Coronal reconstruction image shows a tortuous tubular cyst with partial obscure wall.

**Figure 2:**
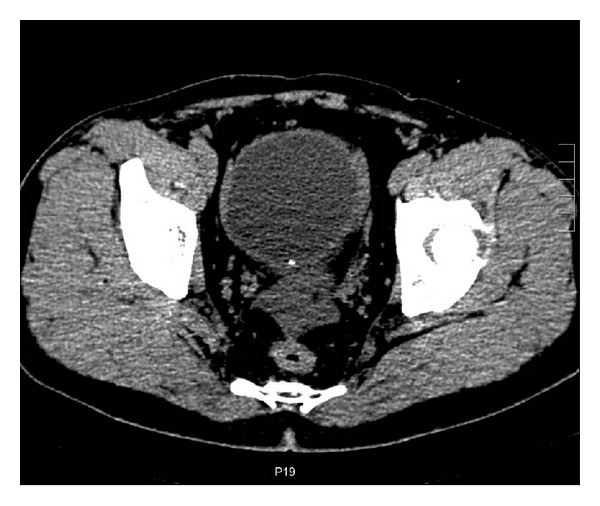
Axial CT shows cystic wall with punctate calcification in the distal portion.

**Figure 3:**
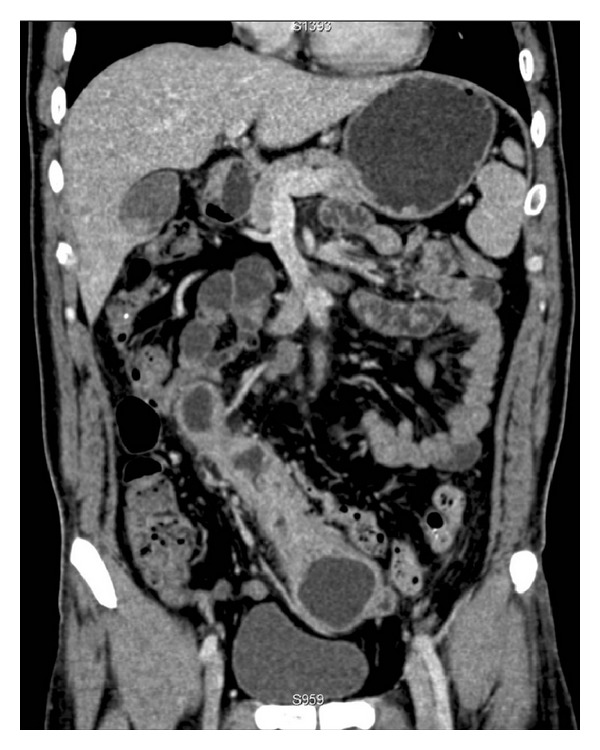
Coronal reconstruction image shows decreased cyst with enhanced wall and absorption of inflammation around it.

A 31-year-old man was admitted to our hospital complaining of abdominal distention and abdominal pain for 1 week. On physical examination, no palpable mass could be found due to abdominal fullness. Laboratory data on admission showed elevated WBC to 10.8 × 10^9^/L with 80% neutrophil. Urinary analysis showed proteinuria 3+, blood 3+. Abdominal ultrasound revealed abdominal cystic mass. Abdominal CT scan showed a giant abdominal cystic mass, which was tortuous and about 27 cm in length and 7.6 cm in width (Figure 1). Haziness of surrounding structure and a minimal pelvic fluid collection suggested existence of inflammation. Punctate calcification was also found in the distal end of the cystic wall (Figure 2). So, antibiotic therapy was administered for one week and the patient's initial symptoms dramatically resolved. Reevaluation of physical examination showed no abnormality. Repeat laboratory data were all within normal range (WBC of 4.9 × 10^9^/L with 56% neutrophil, and urinary analysis normal). Reexamination of abdominal CT showed marked decrease in the size of the cystic mass, the increased thickness of the cystic wall, and the absorption of inflammatory reaction surrounding the cyst (Figure 3).

**Figure 4:**
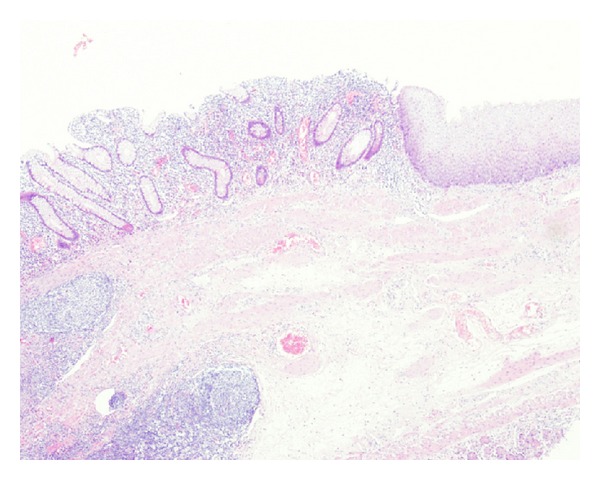
The resected specimen shows ileal mucus gland and esophageal squamous epithelium (HE ×40).

Exploratory laparotomy was necessary for further clarification. Intraoperative finding was a cystic mass in abdominal cavity along the mesenteric border of ileum situated 20 cm proximal to the ileocecal valve, measuring about 20 cm × 5 cm in size. The cystic mass was easily dissected from the distal portion to the proximal portion and was removed together with the adjacent ileum. The proximal portion of the cystic mass was blind end and the distal portion showed communication with the ileum. The final histopathological outcomes were consistent with ileal duplication with ectopic esophageal epithelium and ileal mucus glands (Figure 4). Postoperative period was uneventful. The patient was discharged from the hospital 2 weeks later and remained asymptomatic throughout a 2-year follow-up period.

## 3. Discussion

Duplication of the gastrointestinal system is rare congenital malformation observed in one out of 25,000 deliveries [4]. This rare congenital anomaly was first described by Fitz in 1884 but was not widely used until it was popularized by Ladd in the 1930s. More than 80% of cases of gastrointestinal duplication present before the age of 2 years as an acute abdomen or bowel obstruction, and they can occur anywhere throughout the alimentary tract from mouth to anus. Duplication can be divided in two types: cystic (90%) and tubular (10%). Approximately 75% of duplication has been reported to be located within the abdominal cavity, whereas the remaining is intrathoracic (20%) or thoracoabdominal (5%). It is possible that duplication at different anatomical sites develops through different mechanisms. Several theories have been suggested to explain the cause of duplication [5]. Of these, persistence of fetal gut diverticula, defect in recanalisation of the solid stage of primitive gut, and partial twining, and the split notochord theories are popular. Histopathologically, intestinal duplications have at least one outer muscular layer together with an inner gastrointestinal mucosal lining, which may be composed of several different types of gastrointestinal mucosa. However, ileal duplication cyst lined by ciliated columnar and squamous epithelium has been rarely reported in the literatures [6, 7]. Here, in our case with tubular ileal duplication, there was coexistence of intestinal columnar and esophageal squamous epithelium in the duplicated segment. 

Most of the adult intestinal duplications are asymptomatic and remain undiagnosed for years. Sometimes, they may result in serious morbidity or mortality if left untreated. Intestinal duplications may present with acute onset or chronic complaints, both are possible presentations. Common findings are nausea, vomiting, abdominal pain, constipation, bowel obstruction, volvulus, palpable mass and bloody stool. Occasionally, the enteric cyst can become necrotic and lead to fistula formation with the adjacent intestine, peritoneum, or other nearby structures [8]. In most cases no significant difference in clinical presentation has been detected between communicating and noncommunicating duplication. Because of their common location at mesenteric border, intestinal duplications may be easily mistaken for mesenteric or omental cyst. Because small intestine is a particularly difficult part of the bowel to visualize with routine endoscopic investigation, imaging studies have an important role in establishing the diagnosis. In barium meal studies, in noncommunicating cysts, the alimentary tact can be seen as compressed and displaced by a mass, whereas in the communicating type, the cyst itself can be observed as being filled with contast. Ultrasonography could be helpful in establishing a preoperative diagnosis but it is more effective for the evaluation of enteric cyst duplication in pediatric patient [9].

Multidetector computed tomography enteroclysis has been described as highly accurate in depicting bowel abnormalities and extraintestinal findings in a large variety of small bowel disorders [10]. Duplication cyst can be recognized on a routine CT scan as smoothly rounded, fluid filled cystic, or tubular structure within slightly enhancing walls in or adjacent to the part of the wall of the alimentary tract. Cystic wall calcification is rarely reported in gastric and duodenal duplications, but this finding is often seen in the small intestinal duplication, as described in our case. Uniqueness of our case different from other cases is that repeated CT examination demonstrated dynamic changes of lesion in the cystic size, cystic wall thickness, and inflammatory reaction surrounding the cystic mass after one week of antibiotic treatment. The initial larger cystic lesion may attribute to the obstruction of cystic communication in the distal portion secondary to infection, while the cyst became small in size after inflammatory absorption. Meantime, this change in the presented case probably elucidates why ileal duplication cyst is difficult to be found on the long term based on the fact that it can change itself. 

Ideal treatment of ileal duplication is total excision, but, if not possible, subtotal excision and/or internal derivation is an another option [11]. The overall outcome of surgery is generally favourable. Imaging studies, especially CT scan, are of vital importance in establishing diagnosis and making surgical plans preoperatively.
